# Supplementary material for: MetaRibo-Seq measures translation in microbiomes
Source: Nat Commun. 2020 Jun 29;11:3268. doi: 10.1038/s41467-020-17081-z (PMC7324362; doi:10.1038/s41467-020-17081-z)
Supplement: Supplementary file 10 — Supplementary Data 7 [file 41467_2020_17081_MOESM10_ESM.zip › File2/Confidence_High_Taxonomy/158746_out.krona.html]

Javascript must be enabled to view this page.

members
magnitude
magnitudeUnassigned
count
unassigned
taxon
rank

158746\_out

7

7
superkingdom
2

phylum
7
1239

186801
7
class

7
order
186802

186803
2
family

841
genus
1

species
1

SRS014979\_contig\_number\_contig-100\_1953.186943
1897015


SRS065504\_contig\_number\_36832
1898203
1
species

31979
5
family


SRS016267\_contig\_number\_contig-100\_15024.15024SRS024549\_contig\_number\_contig-100\_1764.31959SRS050422\_contig\_number\_contig-100\_13904.44363SRS063518\_contig\_number\_contig-100\_576.218307SRS105153\_contig\_number\_contig-100\_31001.31001
1485
5
genus
